# The effect of bilingualism on brain development from early childhood to young adulthood

**DOI:** 10.1007/s00429-020-02115-5

**Published:** 2020-07-20

**Authors:** Christos Pliatsikas, Lotte Meteyard, João Veríssimo, Vincent DeLuca, Kyle Shattuck, Michael T. Ullman

**Affiliations:** 1 School of Psychology and Clinical Language Sciences, University of Reading, Reading, RG6 6AL UK; 2 Facultad de Lenguas y Educación, Universidad Nebrija, Madrid, Spain; 3 Potsdam Research Institute for Multilingualism, University of Potsdam, Potsdam, Germany; 4 School of Psychology, University of Birmingham, Birmingham, B15 2SA UK; 5 Departments of Neuroscience and Neurology, Georgetown University, Washington, DC USA; 6 Department of Neuroscience, Georgetown University, Washington, DC 20057-1464 USA

**Keywords:** Bilingualism, Brain development, Grey matter, White matter, Generalized additive models

## Abstract

Bilingualism affects the structure of the brain in adults, as evidenced by experience-dependent grey and white matter changes in brain structures implicated in language learning, processing, and control. However, limited evidence exists on how bilingualism may influence brain development. We examined the developmental patterns of both grey and white matter structures in a cross-sectional study of a large sample (*n* = 711 for grey matter, *n* = 637 for white matter) of bilingual and monolingual participants, aged 3–21 years. Metrics of grey matter (thickness, volume, and surface area) and white matter (fractional anisotropy and mean diffusivity) were examined across 41 cortical and subcortical brain structures and 20 tracts, respectively. We used generalized additive modelling to analyze whether, how, and where the developmental trajectories of bilinguals and monolinguals might differ. Bilingual and monolingual participants manifested distinct developmental trajectories in both grey and white matter structures. As compared to monolinguals, bilinguals showed: (a) more grey matter (less developmental loss) starting during late childhood and adolescence, mainly in frontal and parietal regions (particularly in the inferior frontal gyrus pars opercularis, superior frontal cortex, inferior and superior parietal cortex, and precuneus); and (b) higher white matter integrity (greater developmental increase) starting during mid-late adolescence, specifically in striatal–inferior frontal fibers. The data suggest that there may be a developmental basis to the well-documented structural differences in the brain between bilingual and monolingual adults.

**Electronic supplementary material:**

The online version of this article (10.1007/s00429-020-02115-5) contains supplementary material, which is available to authorized users.

## Introduction

### Bilingualism and brain structure in adults

Recent years have seen an emerging interest in the effects that learning and using more than one language have on brain structure (Luk et al. 2020). An increasing number of studies suggest that, as compared to monolinguals, bilingual (or multilingual) *adults* show structural alterations in cortical regions and subcortical grey matter structures, as well as in white matter tracts that connect these regions (for recent reviews, see Hayakawa and Marian 2019; Pliatsikas 2019). These findings suggest that bilingualism/multilingualism (referred from here onwards as bilingualism) is on par with other types of longer term experience that lead to significant structural brain changes during the acquisition and maintenance of a new skill, such as juggling, the use of novel tools, and navigation (Maguire et al. 2000; Draganski et al. 2004; Quallo et al. 2009; Taubert et al. 2010).

Not surprisingly, such structural changes are generally found in brain regions that are involved in the skill at hand (Maguire et al. 2000; Draganski et al. 2004; Quallo et al. 2009; Taubert et al. 2010). Therefore, structural changes associated with bilingualism may be expected in (at least) those brain structures involved in bilingualism. Bilingualism entails the learning of the knowledge and skills involved in the use of the languages (including phonology, lexico-semantics, and grammar), as well as the (apparently constant) control between them (Green and Abutalebi 2013). Thus, bilingualism may be expected to affect the structure of cortical and subcortical regions involved in (perhaps among other functions) language learning, processing, and control, as well as the white matter tracts that provide connectivity between these regions.

Indeed, the reported structural effects of bilingualism in adults (measured as differences between bilingual and monolingual groups, or sometimes in training studies) are most commonly reported in grey matter regions that have been found to underlie such language-related (as well as other) processes (for details, see Pliatsikas 2019). These regions primary include: frontal and nearby cortex, including the three portions of the left inferior frontal gyrus (IFG), namely, opercularis (IFGop), triangularis (IFGtr), and orbitalis (IFGor), as well as the frontal pole, the middle and superior frontal gyri (MFG and SFG), and the anterior cingulate cortex (ACC); temporal cortex, including the superior, middle, and inferior temporal gyri (STG, MTG and ITG), Heschl’s gyrus, the temporal pole, and the hippocampus; and parietal cortex, including the supramarginal gyrus, the angular gyrus, and the superior parietal lobule (Mechelli et al. 2004; Mårtensson et al. 2012; Abutalebi et al. 2014a; Klein et al. 2014; Stein et al. 2014; Kaiser et al. 2015; Olulade et al. 2016; Hämäläinen et al. 2018). Subcortical structures that are affected mainly include the basal ganglia, in particular the caudate nucleus, the putamen, and the globus pallidus, as well as the thalamus (Burgaleta et al. 2016; Pliatsikas et al. 2017; DeLuca et al. 2019a), with some effects also having been reported in the cerebellum (Filippi et al. 2011, 2020; Pliatsikas et al. 2014). Moreover, several white matter tracts that provide connectivity between these structures (among others) have also been found to be modified by bilingualism. These primarily constitute the inferior and superior longitudinal fasciculi (ILF and SLF), the inferior fronto-occipital fasciculus (IFOF), the arcuate and uncinate fasciculi, and the anterior thalamic radiation, as well as the corpus callosum, including the forceps major and the forceps minor (Luk et al. 2011; Pliatsikas et al. 2015; Kuhl et al. 2016; Mamiya et al. 2016; Rossi et al. 2017; Anderson et al. 2018; Singh et al. 2018).

The specific structural effects of bilingualism within adults appear to vary as a function of both experience and age. In young adult bilinguals with limited experience using their second language (L2) (e.g., with limited immersion in second language speaking environments), studies have typically found cortical tissue increases, reflected as increased cortical thickness and/or cortical volumes (we are not aware of any research examining surface area differences as a function of bilingualism in adults). Such increases have been observed in multiple frontal, temporal, and parietal regions in bilinguals as compared to monolinguals (Mechelli et al. 2004; Ressel et al. 2012; Klein et al. 2014; Olulade et al. 2016) as well as in younger adults in language training studies, at least in initial stages (Mårtensson et al. 2012; Bellander et al. 2016). In contrast, young adult bilinguals with limited L2 experience often show limited or no changes in subcortical volumes (Pliatsikas et al. 2017). Rather, subcortical differences seem to be found in more experienced bilinguals (in particular, those with substantial L2 immersion, or in bilingual environments), who show greater volumes than monolinguals in a variety of subcortical structures, especially in the basal ganglia (in the caudate, putamen, and globus pallidus), as well as the thalamus and the cerebellum (Burgaleta et al. 2016; Pliatsikas et al. 2017; DeLuca et al. 2019b). Similarly, white matter effects have not been reported in young adults with limited L2 experience. Instead, experienced bilinguals (again, especially those with substantial immersion/in bilingual environments) seem to show white matter differences, as compared to monolinguals, in particular increases in white matter integrity (generally reported as increases in fractional anisotropy [FA] and/or decreases in mean diffusivity [MD]; see below for definitions) (Pliatsikas et al. 2015; Rahmani et al. 2017). Experienced bilingual young adults, by contrast, generally do not show differences in cortical thickness or volume as compared to monolinguals (Pliatsikas 2019). Somewhat different patterns have been observed in older (and typically longer term) bilingual adults, who generally show (in comparison to age-matched monolinguals) greater grey matter volumes in similar cortical as well as subcortical regions, in addition to greater white matter integrity, again mainly in the same tracts that are found to change in younger adult bilinguals (Luk et al. 2011; Abutalebi et al. 2014a, b; Olsen et al. 2015; Anderson et al. 2018; Borsa et al. 2018; Del Maschio et al. 2019).

What may explain these patterns? A detailed review of the neuroplastic effects of bilingualism (see Pliatsikas 2019) is beyond the scope of this paper. However, the available models that attempt to explain such effects and their underlying mechanisms have certain common denominators, in addition to the claim that any changes result from the bilingual experience (Li et al. 2014; Hernandez et al. 2015, 2018; Abutalebi and Green 2016; Grundy et al. 2017). Based on these accounts, and in addition to the expansion–partial renormalization hypothesis (EPH), which is designed to explain experience-dependent adult grey matter changes in general (Lövdén et al. 2013), Pliatsikas (Pliatsikas 2020) proposed the dynamic restructuring model (DRM) of structural changes due to bilingualism.

First of all, according to the EPH (on which Pliatsikas’s DRM is based), the acquisition of a new skill is marked by transient local expansions in the implicated grey matter regions, reflected as increases in cortical thickness or volume (such effects on cortical surface area are not discussed in Lövdén et al. 2013). These grey matter tissue increases are posited to be likely due to the development of new dendritic spines (perhaps as well as neurogenesis). The increases are followed by a slow process of pruning older and idle spines, eventually leading to the gradual reversal of the initial expansions (perhaps even back to baseline), as the newly formed networks become more efficient over time and with practice. Putting this into the context of bilingualism, Pliatsikas (2020) suggested that the local grey matter increases reported in relatively inexperienced bilinguals, including those at earlier stages of learning in language training studies, reflect an initial tissue increase in brain structures related to language learning and use. As language use becomes more efficient, these structures are posited to gradually return to baseline. Together, this may explain the patterns described above: cortical thickness and volume increases in less experienced bilinguals (vs. monolinguals) and at early stages of language training in young adults, as well as a lack of such differences in more experienced bilinguals. The emergence of larger subcortical volumes in younger adult bilinguals with greater experience, in particular in the basal ganglia, may be due to their ongoing roles in language control as well as later stages of language learning (proceduralization; Ullman, 2016, 2020); interestingly, in both of these cases one may expect gradual increases as well as eventual (but much later) decreases, as language control becomes more efficient and as more of the language is proceduralised.

Age is also posited to contribute to at least some of the observed structural changes in adult bilinguals. The greater grey matter cortical and subcortical metrics (thickness, volumes) in older adult bilinguals, as compared to older monolinguals (see above), is usually interpreted as greater age-related tissue *loss* for monolinguals, as a result of “strengthened” local connections becoming more resilient to ageing, which might help explain why these effects in older adults only emerge in experienced bilinguals (Perani and Abutalebi 2015). Much less attention, however, has been devoted to *possible age effects during child development*, even though much of the bilingual literature described above relates to additional languages learned (at least part) before early adulthood. Nevertheless, it has been suggested that at least some of the grey matter increases observed in adult bilinguals, as compared to monolinguals, might be explained by less pruning (less grey matter tissue loss) in bilinguals during *brain development*, even though neural mechanisms for this process were not discussed (de Bot 2006).

We now turn to why white matter changes with experience. In general, skill acquisition has been found to lead to increases in white matter integrity (reflected in higher FA or lower MD), which have been interpreted as optimized communication (Scholz et al. 2010). Neurobiologically, white matter integrity increases have been associated with greater myelin in the implicated tracts (Takeuchi et al. 2010). Myelin increases can manifest in different ways (Scholz et al. 2010), one of the most common and important being increased axonal myelination, which is of particular relevance for tract integrity. Axonal myelination depends at least in part on the electrophysiological activity of the axon, in that the more active the axon is the more myelinated it becomes, to provide efficient neural communication (Ishibashi et al. 2006). In this respect, those axons that are more active, e.g., that are involved in the acquisition or use of a new skill, may be expected to be associated with greater myelination, which may in turn be observed as greater white matter integrity. In other words, white matter integrity is at least in part a function of experience, and can be altered due to acquiring and/or using a skill (Zatorre et al. 2013).

Again putting this into the context of bilingualism, in the DRM, Pliatsikas (2020) argued that increases in white matter integrity should generally emerge in more experienced young adult bilinguals, as has been observed. This could be due to increased optimization of different sorts, including proceduralization and various aspects of language use and control. Along these lines, since bilingualism is a life-long experience (at least for those with substantial L2 immersion or in bilingual environments, who show the clearest white matter changes; see above), the increased integrity might persevere in older age and counteract neurodegeneration, giving rise to the effects reported in older bilinguals (Perani and Abutalebi 2015). We are not aware of any theoretical suggestions that adult bilingual/monolingual differences in white matter integrity can be attributed to bilingual/monolingual differences in how the brain *develops* during childhood and adolescence.

In sum, the evidence (and the DRM) suggests that, at least in adults, bilingualism-induced neuroplasticity cycles dynamically through stages. Specifically, relatively early regional cortical grey matter increases likely related to initial stages of language learning, use, or control later proceed to cortical grey matter renormalization, subcortical grey matter increases, and increases of white matter integrity, all possibly related to progressively more efficient language learning, use, or control. However, the role of bilingualism in the *development* of grey and white matter structures prior to adulthood in explaining the observed patterns in *adults* has, as of yet, barely been discussed. Moreover, as we will see below, there has also been very little (empirical or theoretical) work on structural brain changes due to bilingualism *within* children and adolescents. This paper addresses these issues.

### Brain development in childhood and adolescence

Before reviewing the available evidence regarding structural effects of bilingualism on the developing brain, it is useful to first discuss the more general patterns of brain development in (typical) childhood and adolescence. A note on terminology: for simplicity, in this paper we use “development”, “changes” and similar terms to refer to all age-related differences found during childhood and adolescence, including in cross-sectional studies, which constitute the majority of the extant literature on this topic. However, we remind the reader that caution is warranted regarding the inference of developmental trajectories from non-longitudinal data.

Grey matter development from early childhood through adolescence has been studied quite extensively (Giedd 2004; Tamnes and Østby 2018; Vijayakumar et al. 2018; Foulkes and Blakemore 2018). Overall, the evidence suggests that grey matter generally (but not always) *decreases* (reflecting developmental grey matter loss) between early childhood and early adulthood, across cortical and subcortical structures. However, the (linear or nonlinear) *trajectories* of these decreases seem to vary as a function of different factors, including which measures are used (volume, thickness, or surface area) and which structures are examined (Fjell et al. 2009). First of all, cortical thickness tends to decrease continuously throughout childhood and adolescence, though with some regional variability (Remer et al. 2017; Tamnes et al. 2017). In contrast, both cortical surface area and cortical volume show clear nonlinear trajectories, with early increases often peaking within the first decade or so, followed by sustained decreases into early adulthood, with the exact shape varying according to the region examined (e.g., peak hippocampal volumes occur somewhat later, around age 15) (Wierenga et al. 2014; Mills et al. 2016; Tamnes et al. 2017). The volumes of subcortical structures seem to show a more varied pattern: whereas some structures show a similar trajectory of increases (peaking around age 15) followed by decreases (e.g., globus pallidus, amygdala, cerebellum), others instead show linear decreases throughout childhood and adolescence to early adulthood (e.g., the caudate nucleus, putamen, and nucleus accumbens, which together form the striatum), though again there is some variability, including as a function of factors such as sex (Tiemeier et al. 2010; Wierenga et al. 2014; Herting et al. 2018). Overall, the mechanisms underlying these different developmental trajectories remain unclear, though various factors likely contribute to them, including the developmental pruning of dendritic and axonal connections as well as increasing myelination (which may be a significant driver of cortical changes in adolescence) (Chechik et al. 1998; Luo and Leary 2005; Whitaker et al. 2016).

With respect to white matter, a substantial literature employing diffusion-weighted imaging has shown continuous increases in white matter integrity from early childhood to early adulthood, generally reflected as increases in FA and/or decreases in MD (Giedd 2004; Qiu et al. 2008; Lebel and Deoni 2018; Lebel et al. 2019). However, these trajectories show variability according to which tract is examined, as well as according to factors such as sex, genetics, and environmental variables (Lebel and Deoni 2018). Evidence points towards increasing myelination during development as an underlying mechanism that may help explain these trajectories, though the exact nature of this process remains unclear (e.g. increased axonal myelination and/or axonal packing, or other factors; for a review, see Beaulieu, 2002).

### Bilingualism and brain development

Even with the increasing interest in the effects of bilingualism on brain structure in adults, there has been very little work examining such effects in children or adolescents, let alone investigating whether *developmental trajectories* of grey or white matter might be affected by bilingualism. This, despite the suggestion that bilingual brain effects in adults may be at least partly explained by developmental bilingual effects (see above; de Bot, 2006), let alone that the influence of bilingualism on brain development is of interest in its own right.

We are aware of four studies of bilingual effects on grey matter in children or adolescents. The first such evidence was presented by Della Rosa and colleagues (2013), who examined 10-year-old children growing up in a multilingual environment. The children were scanned twice, 12 months apart. The study examined changes specifically in cortical volume, and focused on the left inferior parietal lobule. Della Rosa et al. reported that individuals’ combined competence of their various spoken languages predicted increases during the time period in the cortical volume of the left inferior parietal lobule, a region that appears to underlie (among other functions) the processing of the meaning and phonology of newly acquired words (Richardson et al. 2010). This is the only longitudinal study on children to date probing aspects of bilingualism or multilingualism on grey matter. However, the study did not include a monolingual control group. Given that 10-year olds typically still learn words even in their native language, which in itself might introduce structural changes (Lee et al. 2007), such a comparison would be necessary to attribute the observed effects to multilingualism.

More recently, Archilla-Suerte and colleagues (2018) compared two groups of bilingual children (mean age across both groups: 9.26 years, range 6–13), who had either balanced or unbalanced proficiencies in their two languages. The effect of age was not examined, and monolinguals were not tested. The study focused on three cortical structures (STG, IFG, and MFG), reporting cortical thickness and surface area, and two subcortical structures (caudate nucleus and putamen), for which volumes were reported. Compared to the unbalanced group, the balanced bilinguals had significantly thinner cortex in the left IFGop and MFG, regions related to language learning, processing, and control (Ullman 2004, 2016; Olulade et al. 2016) and in the left transverse STG, which includes Heschl’s gyrus and plays a role in learning non-native sounds (Golestani 2014). The balanced bilinguals also showed significantly larger volumes of the putamen, a structure related to articulatory control (Pliatsikas et al. 2017) as well as to learning in procedural memory and procedural-based aspects of language (Ullman et al. 2020). No significant differences were found between the two groups for cortical surface area. Archilla-Suerte et al. (2018) proposed that their findings are related to bilingualism, but they were uncommitted as to whether brain morphology droves the proficiency differences, or vice versa. Viewed from an experience-based perspective, such as that provided by the DRM, these effects might signify gradual renormalization of previously expanded cortical structures for balanced (and potentially more experienced) bilinguals, accompanied by significant expansion of the putamen—similar to patterns observed in experienced bilingual adults (see above). In contrast, the unbalanced (and presumably less experienced) bilingual children may still be at the stage of initial cortical reorganization that involves increases in cortical thickness.

In another study that tested effects of bilingualism on grey matter, Brito and Noble (2018) compared cortical thickness and surface area between monolinguals and bilinguals, aged between 3 and 21 years. Several covariates were included in their analyses, including age, sex, socioeconomic status, and genetic ancestry. Cortical volumes, as well as subcortical structures, were not examined. The study focused on left IFG, left and right MFG, left STG, and ACC. No main effect of bilingualism (vs. monolingualism) was reported. They additionally examined the effect of bilingualism separately in groups of younger (3–11 years) and older (12–21 years) individuals. The potential effect of bilingualism on developmental trajectories of brain structures was not examined (e.g., no direct comparison of bilingual effects between the age groups was reported). No effect of bilingualism was found in the younger age group. However, in the older group, bilinguals showed greater surface area in the ACC, a region that is central to language control (Abutalebi and Green 2016). Brito and Noble (2018) interpret their findings as suggesting that there might not be robust structural effects of bilingualism in younger children, though it is important to emphasize that averaging across such a large age range (3–11) may obscure developmental patterns.

Finally, Thieba, Long, Dewey and Lebel (2019) compared 3-to-5-year-old children raised in a multilingual environment to children raised in a monolingual environment. The two groups were matched on age, sex, and both maternal education and household income (measures of socioeconomic status). Age effects were not examined. They focused (bilaterally) on three cortical regions of interest (IFGop, IFGtr, IFGor), and moreover performed exploratory analyses on 31 additional cortical (sub)regions, again bilaterally. In all regions, they examined cortical thickness, surface area, and volume. Subcortical structures were not probed. None of their findings survived statistical correction, though in their uncorrected results they reported thicker cortex in the left IFGop and the right caudal MFG, as well as larger volumes in the left caudal ACC, the left caudal MFG, and the right MTG, in the multilingual group as compared to the monolingual group. It is worth noting that the increased cortical thickness in the IFGop and MFG matches the finding of increased cortical thickness for unbalanced (vs. balanced) bilinguals in the same two structures in Archilla-Suerte et al. (2018). This overlap seems consistent with the fact that the participants in Thieba et al. were much younger, and, therefore, had relatively little bilingual experience, just as might be the case for the unbalanced (vs. the balanced) older bilingual children in Archilla-Suerte et al. Therefore, both groups might have been at an earlier stage of bilingualism-induced neuroplasticity in which increased cortical thickness is observed.

The dearth of evidence regarding the effect of bilingualism on brain development is particularly evident in the very limited literature on white matter. We are aware of only two studies on this topic. Mohades and colleagues (2012) compared three groups of 8–11-year-old children: simultaneous bilinguals, sequential bilinguals, and monolinguals. The groups had similar age and sex distributions. The effect of age was not examined. The study focused on FA in four tracts (IFOF, SLF, and two bundles related to the corpus callosum). They reported greater FA values in the left IFOF (which plays important roles in language) for simultaneous bilinguals compared to both sequential bilinguals and monolinguals, as well as in one of the corpus callosum bundles (connecting orbital frontal cortex bilaterally) for monolinguals compared to the two bilingual groups. Interestingly, and despite not emerging as significantly different, the FA values in the left IFOF for sequential bilinguals were intermediate between the values of the two other groups. Additionally, the same participants were scanned again after 2 years (Mohades et al. 2015), in a longitudinal design. The study reported that while all groups showed a significant increase in their FA values in the left IFOF over the 2 years (as expected, given general developmental increases in white matter integrity; see above), the increase was larger for the sequential bilinguals than the other two groups. This appears to be consistent with experience-based increases in white matter integrity due to the bilingual experience. Note that the finding that a larger increase was observed for sequential than simultaneous bilinguals might be partly due to the latter having undergone earlier increases due to their experience.

In sum, there is an emerging literature on the effects of bilingualism on brain structure during development. The findings reveal intriguing overlap both between different developmental groups (e.g., younger bilinguals and somewhat older unbalanced bilinguals), as well as between developmental and adult bilingual effects (see Sect. Bilingualism and brain structure in adults). Overall, the results suggest that similar experience-based effects may be found during development and in adults, and that developmental patterns might in fact help explain adult patterns.

Nevertheless, a number of important gaps remain, including the following. First and perhaps most importantly, there is a dearth of research comparing the developmental trajectories of brain structures between bilinguals and monolinguals, that is, examining interactions between bilingualism/monolingualism and age, including nonlinear effects. Indeed, we are not aware of any studies probing this issue over the course of childhood and adolescence. Such studies seem critical for understanding how development may lead to bilingual effects in adults, let alone for understanding how bilingualism affects the course of development. Second, not all studies have controlled for certain potentially confounding variables (e.g., sex, socioeconomic status). Third, most previous developmental studies probing bilingual effects prior to adulthood have focused on specific brain structures: generally particular grey matter cortical regions, leaving subcortical structures and white matter greatly understudied. Fourth, prior studies have also focused on particular measures that moreover often differ between studies (e.g., only volume, or only cortical thickness and surface area, or only FA). Indeed, we are not aware of any research that has widely examined both grey and white matter within subjects, let alone with all major measures (cortical thickness and surface area, volumes, FA, and MD). Thus, our understanding of the potential effects of bilingualism on brain development is still quite limited. This gap seems to warrant clarification with a more comprehensive study.

### The present study

The present study was designed to address these gaps. We analyzed a large existing dataset of children and adolescents (PING, Jernigan et al. 2016), aged 3–21. The dataset included both bilinguals and monolinguals, who were reasonably evenly distributed across the age range. To comprehensively investigate the effect of bilingualism on brain development, we directly compared the two groups’ developmental trajectories in a wide range of brain structures. We controlled for a number of potentially confounding variables, including sex, genetic ancestry, and socioeconomic factors (both parental education and household income) (Bakken et al. 2011; Noble et al. 2015; Fan et al. 2015; Romeo et al. 2018; Foulkes and Blakemore 2018). We examined effects bilaterally in a wide set of (both cortical and subcortical) grey matter structures and white matter tracts. For grey matter, we probed cortical thickness, surface area, and volume (with the latter also examined for subcortical structures), while for white matter we examined both FA and MD.

If bilingualism indeed has somewhat analogous effects on the brain in children and adolescents as in adults, then one might expect similar grey and white matter effects of bilingualism during development as in adults, in similar structures, though (in some manner) *overlaid or interacting* with more general developmental brain trajectories (which are described in Sect. Brain development in childhood and adolescence). Thus, we might expect the following: (a) For cortical grey matter, the increases in cortical thickness and volumes observed in adult bilinguals at earlier stages of the bilingual experience (in training studies or as compared to monolinguals), followed by gradual decreases in more experienced bilinguals, may be reflected during development as initial increases followed by gradual decreases over the course of childhood and adolescence. These patterns should *overlap* with the general developmental trajectories of continuous decreases in cortical thickness, and nonlinear increases followed by decreases in cortical volume. Thus, the expected bilingual vs. monolingual “increases” would manifest as less steep age-related decreases (less grey matter loss) in cortical thickness, and perhaps volume as well, with these differences eventually disappearing as the trajectories of the two groups gradually converge. The dearth of adult bilingual evidence from cortical surface area precludes clear bilingual vs. monolingual developmental predictions for this measure. (b) By contrast, and again based on the adult bilingual literature, during the course of development we might expect increases in subcortical volumes in bilinguals as compared to monolinguals, but only after bilinguals have experienced a substantial period of bilingualism, perhaps around the same period that bilingual/monolingual differences in cortical thickness and volume cease to be observed. Again, this pattern should overlap with overall developmental trajectories for subcortical structures. (c) Similarly, greater white matter integrity in bilinguals as compared to monolinguals may be expected to emerge only at higher levels of bilingual experience during childhood or adolescence (perhaps around the same time as the subcortical bilingual/monolingual differences emerge), again overlapping the general developmental pattern of increases in white integrity (as measured by FA and/or MD).

In sum, it seems reasonable that over the course of development, one might expect the following: Bilinguals should show increasing cortical thickness and volumes as compared to monolinguals (less steep decreases, that is, less grey matter loss), with no or few concurrent group differences in either subcortical volumes or white matter. In contrast, subcortical volumes and white should show bilingual effects (larger subcortical volumes and increased white matter integrity as compared to monolinguals) mainly at later stages of experience, at about the same time that cortical thickness and volume differences are no longer observed.

## Methods

### Participants

This study analyzed the dataset provided by the Pediatrics, Imaging, Neurocognition and Genetics (PING) project (Jernigan et al. 2016) (also examined in Brito and Noble 2018). PING is a multi-site collaborative repository comprising demographic, neuroimaging, medical, and cognitive data from 1493 typically developing children, adolescents, and young adults aged between 3 and 21 years. Neuroimaging data for grey matter metrics were available from 1293 participants, and for white matter metrics from 1119 participants. Because this study was concerned with the brain structure and language background of typically developing individuals, we excluded participants with a diagnosis or personal history of ADHD, learning problems, hearing problems, speech and language therapy, meningitis, seizures, head injury, or “unconsciousness”. Moreover, we restricted our analysis to participants that reported English as their first language (see below). Any participants with missing data points for key variables in our models (age, sex, parental education, household income, genetic ancestry, scanner site, as well as language background), were excluded. This resulted in final samples of 711 participants with grey matter data and 637 with white matter data. Finally, the participants were split into groups according to whether they spoke language(s) in addition to English (bilinguals), or not (monolinguals); see below. Demographic and other participant-level information for the final sample are provided in Table 1.

**Table 1:** Participant demographic and related information

	Grey matter		White matter	
	Bilinguals	Monolinguals	Bilinguals	Monolinguals
*n*	141	570	127	510
*n (by age range)*				
3–6	13	97	11	84
6.1–10	42	171	37	154
10.1–14	24	136	20	125
14.1–18	33	97	31	89
18.1–21	29	69	28	58
Mean age (SD)	12.48 (5.11)	11.15 (4.87	12.73 (5.11)	11.15 (4.79)
Females/males	77/64	283/287	67/60	251/259
*Parental education level*				
1 = Less than 7 years of school	4	0	4	0
2 = 7–9 years of school	2	2	2	2
3 = 10–11 years of school	2	8	0	7
4 = High school graduate	10	61	9	58
5 = 1–3 years of college (also business school)	33	147	31	130
6 = 4-year college graduate	33	164	30	148
7 = Professional degree	57	188	51	165
*Household income*				
1 = < $5,000	8	14	6	13
2 = $5,000—9,999	3	20	3	18
3 = $10,000—19,999	6	37	5	33
4 = $20,000—29,999	6	44	6	42
5 = $30,000—39,999	10	41	9	37
6 = $40,000—49,999	10	33	8	29
7 = $50,000—99,999	45	164	43	144
8 = $100,000—149,999	23	120	20	110
9 = $150,000—199,999	12	53	9	45
10 = $200,000—249,999	10	15	10	12
11 = $250,000—299,999	4	12	4	12
12 = $300,000 +	4	17	4	15

For more information on each of the variables above (e.g., parental education level, household income) see Akshoomoff et al. (2014); Jernigan et al. (2016). The age bands are provided for informational purposes; all analyses included age as a (nonlinear) continuous variable

## Data description

### Background participant information

A number of measures of key background characteristics (see "Introduction" and Table 1) were used as covariates in the analyses: sex (male/female); scanner site (which of the thirteen scanners employed in the PING data was used for the particular participant); socioeconomic status, included both as level of household income, provided in twelve bands in PING, as well as highest parental education level, provided in seven bands (see Table 1); and genetic ancestry of the participants, provided by PING in the form of six Genetic Ancestry Factors (proportion of European, African, American Indian, East Asian, Oceanian, and Central Asian ancestry for each participant).

The background questionnaire that was administered as part of PING included the following two questions related to language use: (a) “*Was English the first language participant learned to speak?*” (Yes/No), and (b) “*Does participant speak a language other than English?*” (Yes/No). Only participants who responded “Yes” to (a) were included. These were subsequently split into bilinguals (potentially including multilinguals) and monolinguals based on their response to (b) (coded in our analyses as bilingualism: yes/no). Note that participants who responded “No” to (a) were excluded, as in this case it was not possible to judge whether they were monolingual or bilingual.

### Brain data

PING provides pre-processed measures of brain morphology obtained from a Freesurfer processing pipeline (Fischl 2012); see Jernigan et al. (2016) for a detailed description of data collection and processing. We analyzed data from all grey matter structures and white matter tracts available in the PING database (see Table 2). For cortical grey matter, PING provides measures of thickness, volume, and surface area for 33 cortical regions, based on the cortical parcellation described in Desikan and colleagues (2006, Table 1). Further, PING includes volumes of eight subcortical structures and the cerebellum (Fischl et al. 2002). With respect to white matter, PING provides measures of fractional anisotropy (FA) and mean diffusivity (MD), as well as of longitudinal (axial) and transversal (radial) diffusivity, for 20 tracts or tract subdivisions (Hagler et al. 2009; Yendiki et al. 2011). In this study, we focus on FA and MD, since longitudinal and transversal diffusivity can be difficult to interpret (Singh et al. 2018), and indeed are often not reported or discussed in studies probing white matter integrity, including in the bilingual literature (but see Singh et al., for adults). Nevertheless, for the sake of completeness and transparency, since these measures were available in PING, we performed the same analyses on these as on the other metrics (see below), and report significant effects in Supplementary Material; we do not discuss these measures further in the paper.

**Table 2:** Brain structures and tracts examined in the present study

Cortical regions	Subcortical structures
Frontal pole	Nucleus accumbens
Orbitofrontal cortex-lateral	Caudate nucleus
Orbitofrontal cortex-medial	Putamen
Inferior frontal gyrus-pars opercularis	Pallidum
Inferior frontal gyrus-pars triangularis	Thalamus
Inferior frontal gyrus-pars orbitalis	Ventral diencephalon
Rostral middle frontal gyrus	Amygdala
Caudal middle frontal gyrus	Hippocampus (volumes only, so treated as subcortical)
Superior frontal gyrus	
Temporal pole	Cerebellum
Inferior temporal gyrus	
Middle temporal gyrus	White matter tracts
Banks of the superior temporal sulcus	Superior longitudinal fasciculus
Superior temporal gyrus	parietal portion
Transverse temporal cortex	temporal portion (arcuate fasciculus)
Inferior parietal cortex	Inferior longitudinal fasciculus
Superior parietal cortex	Inferior frontal—Superior frontal cortex
Supramarginal gyrus	Inferior fronto-occipital fasciculus
Rostral anterior cingulate cortex	Striatal—Inferior frontal cortex
Caudal anterior cingulate cortex	Uncinate fasciculus
Posterior cingulate cortex	Anterior thalamic radiation
Isthmus cingulate cortex	Cingulate cingulum
Entorhinal cortex	Parahippocampal cingulum
Parahippocampal gyrus	Cortico-spinal/pyramidal
Precentral gyrus	Superior cortico-striate
Postcentral gyrus	frontal portion
Paracentral gyrus	parietal portion
Cuneus	Fornix
Precuneus	Fornix excluding fimbria
Fusiform gyrus	Corpus callosum
Lateral occipital cortex	Forceps major
Lingual gyrus	Forceps minor
Pericalcarine cortex	

Structure and tract nomenclature follow those used in the PING dataset (Jernigan et al. 2016). Tract subdivisions are indented

### Data analysis

Data were analyzed in R (R Core Team 2014) with generalized additive models (GAMs), using the bam() function in the mcgv package (Wood 2011). GAMs provide the means to fit a nonlinear regression spline, that is, a ‘wiggly’ curve that consists of the sum of simpler nonlinear functions. Additionally, GAMs prevent overfitting the data by penalising such wiggliness, which is only included when there is sufficient evidence for a particular shape. For these reasons, GAMs are well suited to model a variety of curved shapes, including the nonlinear patterns observed in brain development as a function of age (Chang et al. 2016; Tamnes et al. 2017). Separate GAMs were run for each structure/tract and each metric: cortical thickness, surface area, and volume for grey matter cortical regions; volume for grey matter subcortical structures and the cerebellum; and FA and MD for white matter.

We applied an analytical procedure akin to using a “vibration of effects” approach (Patel et al. 2015). This approach involves running a number of alternate models in which parameters are slightly changed across them. Reliability is assessed in terms of consistency across the models; that is, effects that are consistent across alternate models can be considered to be reliable. In GAMs, the estimation of nonlinear effects, and in particular of nonlinear ‘difference curves’ (for example, the difference between the effect of age for monolinguals and bilinguals) can be sensitive to the particular specification of parameters in the model, specifically, to what level is chosen as the reference. Thus, in the present study, the comparison reference level for key variables of interest (bilingualism, hemisphere) was changed to generate a set of similar models. For example, for the first-level model (see just below), the GAM for each bilateral structure/tract and metric was run four times, with all combinations of bilingualism (yes/no) and hemisphere (left/right) as reference levels. Following a conservative approach (to minimise type I errors), effects of interest were assessed as reliable only if they were significant across all of these models (*p*s < 0.05), and thus were not dependent on which reference level was used for comparison.

For the first-level model ('Model 1' in the ‘Code for data analysis’ in Supplementary Material), for each bilateral structure/tract (that is, all apart from the corpus callosum and forceps minor and major) and each metric, we first applied a GAM in which we fit a regression spline for the effect of age (for the group and hemisphere that were selected as reference levels), as well as the difference curves corresponding to the age x bilingualism interaction, the age x hemisphere interaction, and the three-way age x bilingualism x hemisphere interaction. This model also included random effects for participant and scanner site, as well as the covariates parental education, household income, and genetic ancestry as linear terms. Sex was not included as a covariate because it was well matched (see Table 1) within each participant group (i.e., for bilinguals and monolinguals, for both the grey and white matter analyses). As described above, this model was run four times (for each structure/tract and metric). If the three-way interaction (age x bilingualism x hemisphere) was significant in all four models, these were followed up by the second-level model (see next paragraph), separately for each hemisphere. If the three-way interaction was not significant in all four models (or if none of the second-level analyses in either hemisphere were significant, following up from a significant three-way interaction), we again ran the second-level model, but analyzing both hemispheres together (as two repeated measures for each participant).

The second-level model (‘Model 2′) included a term for bilingualism (as an ordered factor, which allows estimating interactions), a regression spline term for age, and a difference curve for the age x bilingualism interaction, as well as all random effects and covariates included in the first model. This allowed us to assess whether the pattern of age-related changes (that is, the developmental trajectory) differed between bilinguals and monolinguals: either in each hemisphere separately (as follow-ups to reliable three-way interactions), or across the two hemispheres (if the corresponding three-way interaction was not reliable). This model was also applied to the corpus callosum, forceps minor, and forceps major, for which the first-level model was not run. In all instances, the second-level model was run twice, once with each level of bilingualism (monolingual and bilingual) as the reference level. It was considered reliable only if the age by bilingualism interaction was significant for both reference levels.

Finally, for structures/tracts and metrics where the age x bilingualism interaction in the second-level model was reliable (significant for both reference levels of bilingualism), a third-level model (‘Model 3′) was run, to unpack this interaction. This model included a main effect of bilingualism, and a regression spline for age for each level of bilingualism (Yes/No) separately.

## Results

As described above, the first-level analysis for all structures/tracts and metrics examined the three-way interaction between age, bilingualism, and hemisphere. In only two cases did these models yield reliable three-way interactions (that is, the model was statistically significant across all four reference levels, as described above): for the volume of the putamen and the surface area (SA) of the posterior cingulate cortex. However, the follow-up second-level analyses for these three-way interactions did not yield any significant effects of bilingualism, that is, either main effects of bilingualism or age by bilingualism interactions, for either hemisphere and for either of these structures and metrics; see Table 3.

**Table 3:** Three-way interactions and their follow-up analyses

	First-level analysis	Second-level analyses			
	Age × Bilingualism × Hemisphere	Left hemisphere		Right hemisphere	
		Bilingualism	Age × Bilingualism	Bilingualism	Age × Bilingualism
Putamen (volume)	**	ns	ns	ns	ns
Posterior cingulate cortex (SA)	*	ns	ns	ns	ns

All edfs (estimated degrees of freedom) > 1. All *F*s  > 3. Only key effects are shown

*ns* not significant, *SA* surface area

Significance level: **ps in all alternate models (four for first-level analyses) < 0.01; *ps < 0.05

**Fig. 1:**
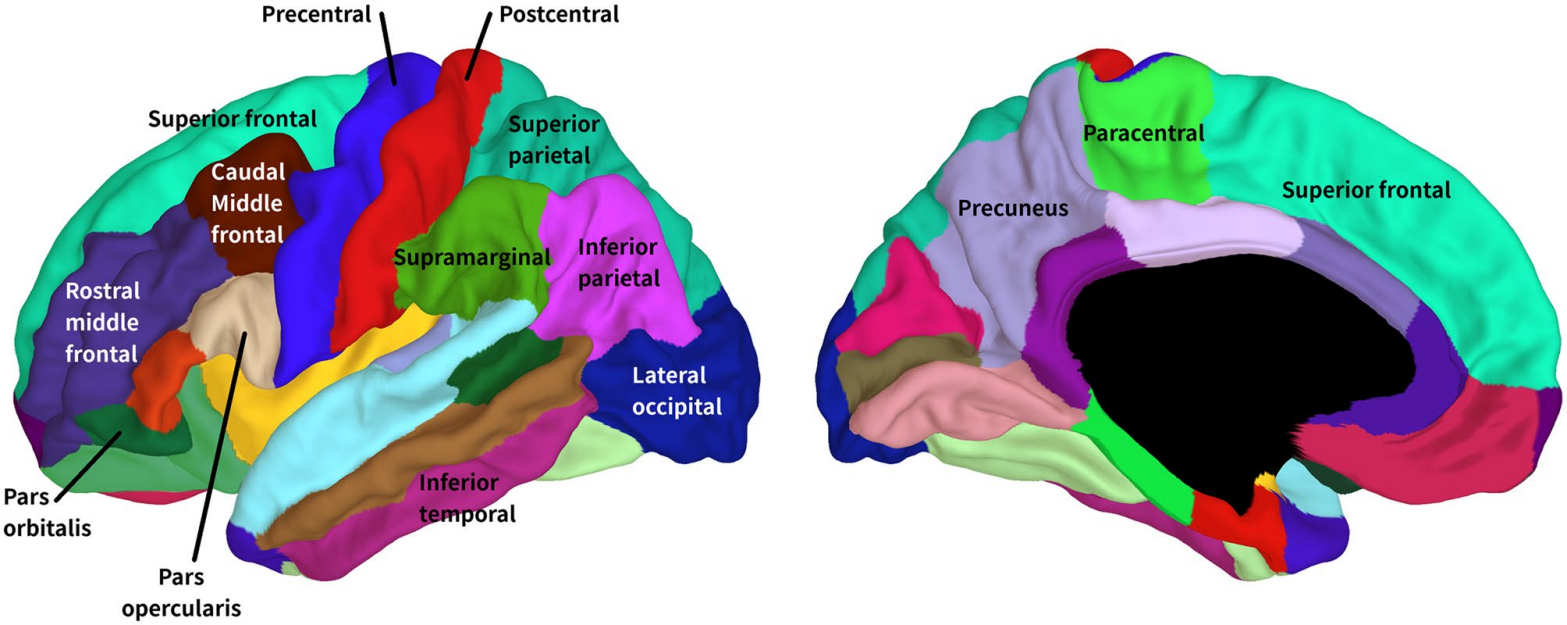
The 14 cortical regions that showed reliable age by bilingualism interactions, that is, for any of the three cortical metrics (cortical thickness, volume, and surface area)

**Fig. 2:**
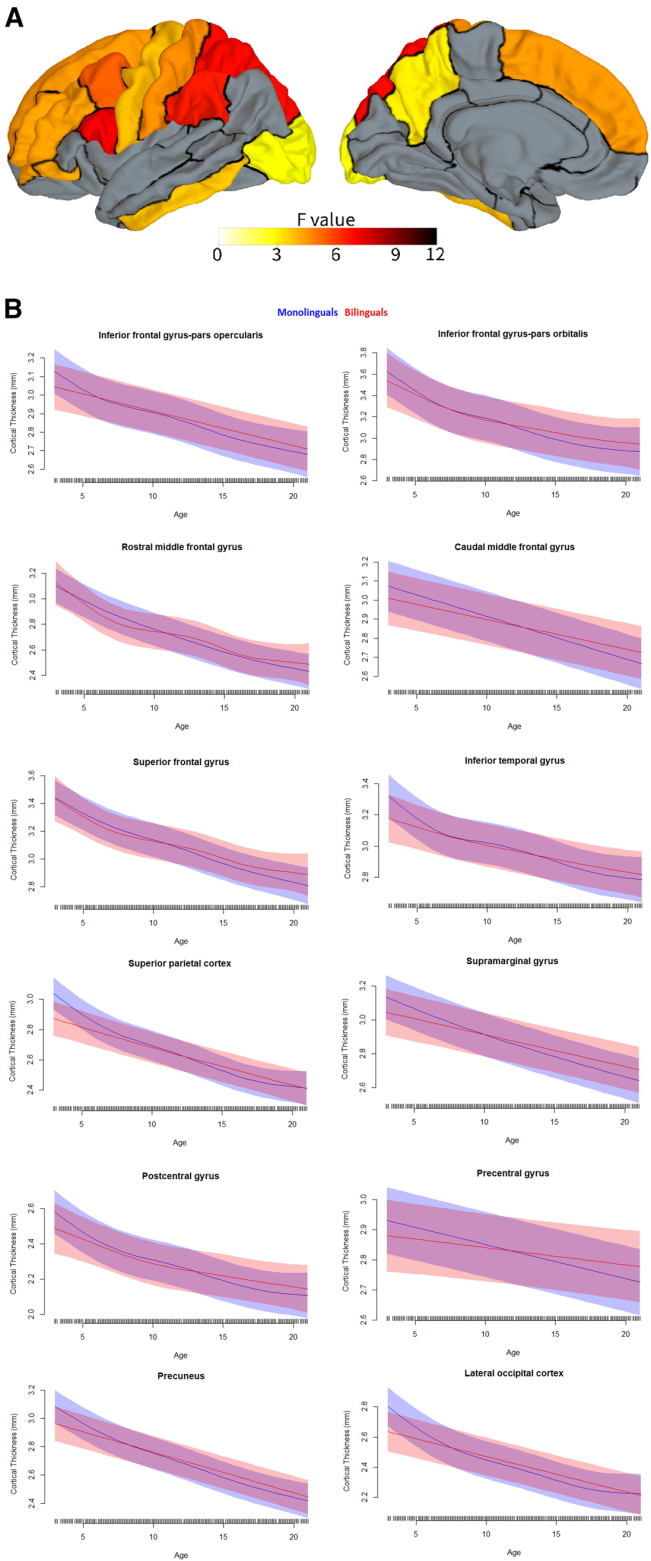
Cortical regions yielding reliable age by bilingualism interactions for cortical thickness. This displays which cortical regions show significantly different developmental trajectories for cortical thickness between age 3 and 21 for bilinguals versus monolinguals. In panel A, the color map reflects *F* values of each interaction between ages by bilingualism (based on the smaller *F* value of the two alternate analyses). In panel B, the developmental trajectories of bilinguals are shown in red, while the trajectories of monolinguals are shown in blue

**Fig. 3:**
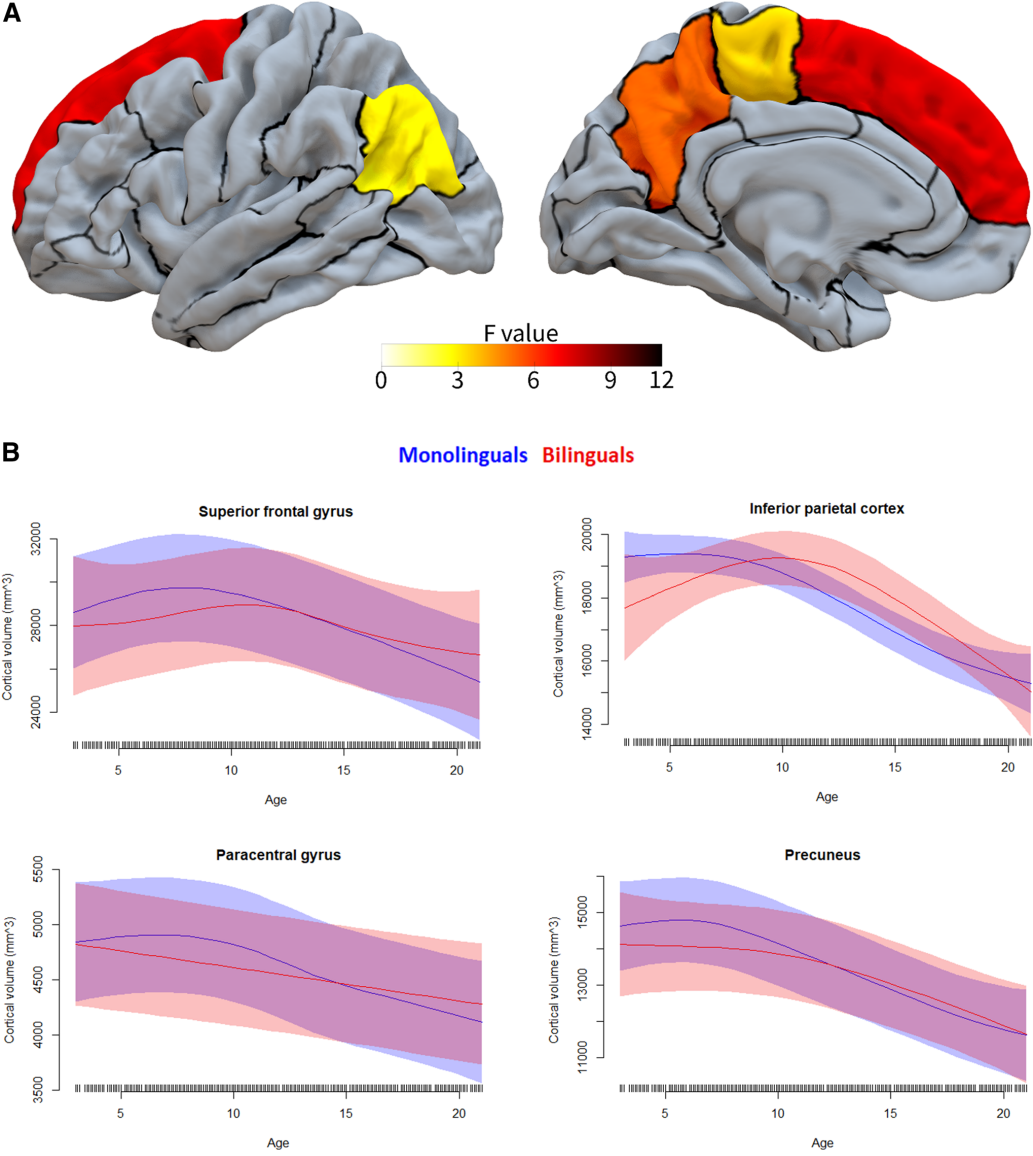
Cortical regions yielding reliable age by bilingualism interactions for cortical volume. This displays which cortical regions show significantly different developmental trajectories for cortical volume between age 3 and 21 for bilinguals versus monolinguals. See Fig. 2 for more information

**Fig. 4:**
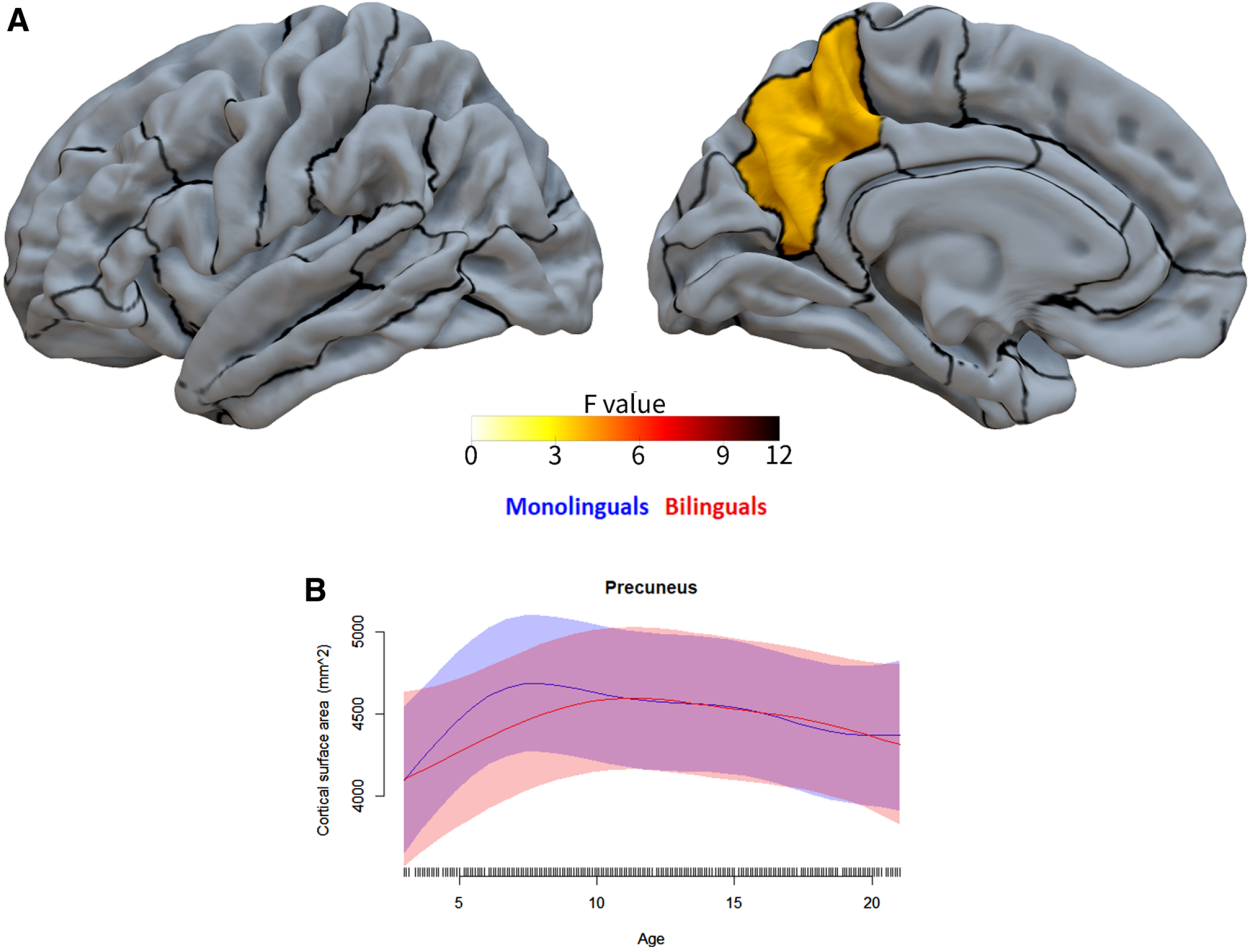
The single cortical region that yielded a reliable age by bilingualism interaction for cortical surface area. This region shows significantly different developmental trajectories for cortical surface area between age 3 and 21 for bilinguals versus monolinguals. See Fig. 2 for more information

**Fig. 5:**
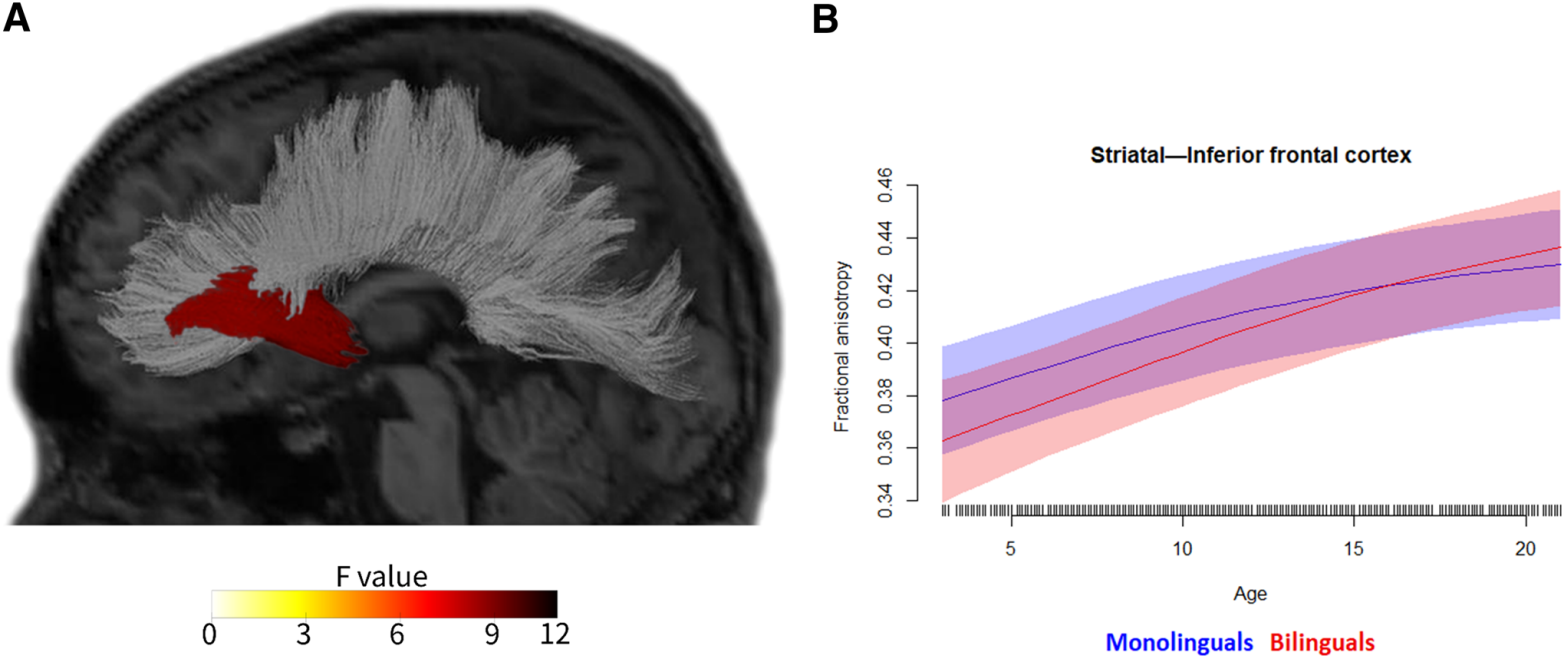
The single white matter tract that yielded a reliable age by bilingualism interaction, specifically for fractional anisotropy. This tract shows significantly different developmental trajectories for FA between age 3 and 21 for bilinguals versus monolinguals. Panel A adapted, with permission, from Reyes et al. (2018)

Following our analysis plan, we then ran second-level models on all structures/tracts and metrics while analysing both hemispheres together (as well as on the corpus callosum, forceps minor, and forceps major), to test for two-way interactions between age and bilingualism, independent of hemisphere. Eighteen of these analyses yielded reliable age by bilingualism interactions (that is, the interaction was statistically significant across both reference levels, as described above). This indicates that in these 18 cases, the developmental trajectories of these metrics for these structures or tracts differed significantly between bilinguals and monolinguals. See Table 4 and Figs. 1, 2. The 18 analyses included 12 cortical regions with cortical thickness as the metric (Table 4, Fig. 2), 4 cortical regions with cortical volume as the metric (Table 4, Fig. 3), 1 cortical region with cortical surface area as the metric (Table 4, Fig. 4), and 1 white matter tract with FA as the metric (Table 4; Fig. 5). Figure 1 labels the cortical regions that showed any reliable age by bilingualism interactions, that is, for cortical thickness, volume, or surface area. Note that a total of 14 cortical regions and 1 white matter tract showed reliable analyses, since 2 cortical regions (the precuneus and superior frontal gyrus) yielded reliable interactions in more than 1 metric; see Table 4. No subcortical structures yielded significant age by bilingualism interactions.

**Table 4:** Two-way interactions and their follow-up analyses

	Second-level analysis		Third-level analyses	
			Bilinguals- Age	Monolinguals- Age
	Bilingualism	Age × bilingualism		
*Cortical thickness*				
Inferior frontal gyrus–pars opercularis	ns	**	***	***
Inferior frontal gyrus–pars orbitalis	ns	*	***	***
Rostral middle frontal gyrus	ns	*	***	***
Caudal middle frontal gyrus	ns	*	***	***
Superior frontal gyrus	ns	*	***	***
Inferior temporal gyrus	ns	*	***	***
Superior parietal cortex	ns	**	***	***
Supramarginal gyrus	ns	*	***	***
Postcentral gyrus	ns	*	***	***
Precentral gyrus	ns	*	*	***
Precuneus	ns	*	***	***
Lateral occipital cortex	ns	*	***	***
*Cortical volume*				
Superior frontal gyrus	ns	**	*	***
Inferior parietal cortex	ns	*	***	***
Paracentral gyrus	ns	**	**	***
Precuneus	ns	*	***	***
*Cortical surface area*				
Precuneus	ns	*	ns	***
Fractional anisotropy				
Striatal—inferior frontal cortex	**	**	***	***

All edfs (estimated degrees of freedom) > 1. All *F*s > 2.5. Only key effects shown (age was a reliable predictor for the vast majority of the second-level analyses)

*ns* not significant

Significance level ***ps (in both alternate models for second-level analyses) < 0.001; **ps < 0.01; *ps  < 0.05

## Discussion

In the present study, we investigated whether and how brain development may be affected by bilingualism—that is, to what extent being bilingual (or multilingual) versus monolingual might influence the developmental trajectories of brain structures during childhood and adolescence. To examine this question, we analyzed a large dataset of individuals, aged 3–21, that included both bilinguals and monolinguals reasonably evenly distributed across the age range (PING) (Jernigan et al. 2016). We analyzed cortical thickness, volume, and surface area for 33 cortical regions, as well as subcortical volumes for 8 structures, for 711 participants; and both fractional anisotropy (FA) and mean diffusivity (MD) for 20 white matter tracts, for 637 participants. The data were analyzed with generalized additive models (GAMs) to reveal the expected nonlinear patterns of brain development while preventing overfitting, while using an analytical procedure akin to a vibration of effects approach to minimize Type I errors.

### Interpretation

Our predictions were based on a combination of previous data and models pertaining to the effects of bilingualism on the brain in adults, together with well-documented general effects of development on the brain. Specifically, we expected that, on top of basic brain developmental patterns, during development greater cortical thickness and volumes (e.g., due to less steep developmental decreases for cortical thickness, that is, less grey matter loss) should emerge for bilinguals as compared to monolinguals, followed by gradual convergence of these metrics between the two groups. (The paucity of adult bilingual evidence from cortical surface area precluded predictions regarding this metric.) In contrast, greater bilingual (vs. monolingual) subcortical volumes and white matter integrity were only expected at later stages of bilingualism, around the same time that cortical differences cease to be observed.

The analyses revealed that while both bilinguals and monolinguals showed the expected general developmental patterns of brain structures, they also showed differences in their developmental trajectories.

First, consistent with the broader developmental literature, both groups showed the following patterns (see Table 4 and Figs. 2, 3, 4, 5). Both evidenced continuous decreases in cortical thickness throughout childhood/adolescence (Remer et al. 2017; Tamnes et al. 2017). In both, cortical volume and cortical surface area generally showed more nonlinear trajectories, often with early increases or stability followed by decreases (Wierenga et al. 2014; Mills et al. 2016; Tamnes et al. 2017). And in both groups, white matter integrity increased continuously with age (Lebel and Deoni 2018; Lebel et al. 2019). Overall, this demonstrates that the patterns observed here follow expected trajectories of brain development.

Second, and of greater interest here, the two groups also showed developmental *differences*, which were moreover strikingly similar across measures and structures (Table 4 and Figs. 2, 3, 4, 5). As expected based on the adult literature (see above), greater cortical thickness and cortical volume (less developmental grey matter loss) were observed for bilinguals as compared to monolinguals across multiple cortical regions, with this deviation becoming apparent around late childhood to early puberty (in most cases about age 10–13 for cortical thickness and 12–14 for cortical volume). Moreover, both for cortical thickness and volume, this pattern continued in most regions; though in some cortical areas the values for bilinguals and monolinguals reconverged in early adulthood, about age 20–21. Additionally, across cortical regions, a very early and unexpected difference between the groups was observed: starting from around age 3, the lowest age in our sample, bilinguals displayed lower cortical thickness and volumes than monolinguals, with these values only converging between the groups from around mid to late childhood to early puberty. However, given the relatively small sample size of the bilingual group at the youngest ages (Table 1), this finding should be treated with caution. Interestingly, though we did not have clear predictions for cortical surface area (due to the dearth of studies examining this metric in bilinguals; see above), the one region showing bilingual/monolingual differences in surface area showed a pattern somewhat similar to that observed for cortical volumes, in particular during early childhood. In sum, the bilinguals consistently showed lower values for cortical metrics than monolinguals until mid to late childhood or early puberty, at which point they generally diverged again in showing larger values (especially for cortical thickness and volume), with this bilingual increase continuing into early adulthood, or in some cases reconverging.

Unlike cortical regions, no differences between bilinguals and monolinguals were observed for subcortical structures or the cerebellum. This null effect, which was consistent with our predictions, adds to the literature in suggesting that subcortical and cerebellar effects may only emerge at later stages in more experienced bilinguals, after the initial cortical effects have started disappearing (Filippi et al. 2011, 2020; Pliatsikas et al. 2014, 2017; Pliatsikas 2020).

With respect to white matter, one tract showed bilingual/monolingual differences, that is, striatal–inferior frontal fibers for FA. Strikingly, this again yielded the same pattern as cortical regions, namely lower FA values from age 3 for bilinguals than monolinguals, with the groups gradually converging, till the bilinguals began to show larger values from about age 16. Overall, this suggests a greater increase for bilinguals than monolinguals in white matter integrity over the course of development.

The pattern of results suggests consistency not only in the developmental trajectories of the different metrics, but also in which parts of the brain show effects. In particular, almost all of the bilingual/monolingual by age interactions were found for frontal or parietal structures. This held across all three cortical metrics (thickness, volume, surface area) and the one implicated white matter tract (which connects the striatum and inferior frontal cortex). Only two regions yielded effects outside these two lobes (one in the temporal lobe, one in the occipital lobe), neither of which were among the strongest interactions observed.

How might the implication of frontal and parietal structures in bilingual/monolingual developmental differences, as well as striatal–inferior frontal structural connectivity, be interpreted? Though interpretation must be made with caution, due to the risk of reverse inference (Poldrack 2006, 2011), this pattern appears to be consistent with the involvement of particular circuits (networks).

First of all, according to certain ‘dual-stream’ views, language and audition (as well as vision) are processed by a ventral stream that is closely related to temporal cortex and underlies aspects of auditory object identification and meaning, together with a dorsal stream that projects from parietal (mainly inferior parietal and nearby cortex) to frontal regions (mainly (pre)motor and IFG pars opercularis) and is primarily involved in sensory–motor integration and articulatory functions (Hickok and Poeppel 2007; Rauschecker and Scott 2009). In the present study, the implication both of inferior parietal regions (supramarginal gyrus and inferior parietal) and motor and related regions (precentral, paracentral, as well as posterior portions of superior frontal and perhaps caudal middle frontal) as well as IFG pars opercularis, thus appears to be consistent with changes in the dorsal stream in the developing bilingual brain. Indeed, the supramarginal gyrus, pars opercularis, and superior frontal gyrus showed particularly reliable differences between developmental trajectories for bilinguals and monolinguals (Figs. 2 and 3). The involvement of the dorsal stream would be consistent with evidence suggesting increased contributions from this stream during at least reading in bilinguals as compared to monolinguals (Parker Jones et al. 2012; Bakhtiari et al. 2014), findings that have been attributed to increased articulatory competition in bilinguals (Parker Jones et al. 2012). A similar argument could also be made for increased competition among the different grammars in bilinguals, particularly given the dependence of grammar on pars opercularis and nearby premotor cortex (Friederici 2011). Nevertheless, bilingual-based developmental changes in the dorsal stream do not seem to full explain the observed patterns, not only because changes in other structures were also observed, but additionally because the supramarginal gyrus showed greater effects than more posterior aspects of inferior parietal cortex, which have generally been the focus in language-related dorsal stream models (Hickok and Poeppel 2007; Rauschecker and Scott 2009).

Second, it is possible that the pattern could (also) be due in part to changes in the procedural memory circuit that appears to underlie language (Ullman 2004, 2020). Both the basal ganglia (in particular the striatum) and frontal cortex (in particular (pre)motor regions and the IFG pars opercularis), as well as parietal regions, play key roles in this circuit, which is implicated in the learning, representation, and use of both first and second language (whereas dorsal stream models focus on first language). Procedural memory may underlie multiple portions of language, including grammar, speech–sound representations, articulation, and more generally aspects of both speech production and speech perception (Ullman 2020; Ullman et al. 2020). Thus, the greater involvement of this system in bilinguals than monolinguals would not be surprising, given the need for all these aspects of language to be supported in two languages in bilinguals, as compared to one in monolinguals. The involvement of the striatal–inferior frontal tract is particularly striking, especially given that it yielded the largest bilingual/monolingual by age interaction (see Fig. 5), that is, the greatest difference in developmental trajectories between bilinguals and monolinguals. Interestingly, given the suggestion that the learning of dorsal stream parieto-frontal circuits may depend importantly on procedural memory (Ullman 2004; Ullman et al. 2020), the findings here may be interpreted as implicating both dorsal stream and procedural memory functions as two sides of the same coin.

Third, the patterns observed here may additionally be explained by the involvement of brain structures underlying executive functions. A large literature in both children and adults has implicated a greater role for executive functions in bilinguals than monolinguals, in particular due to the switching of and the control between languages in bilinguals (Valian 2015; Abutalebi and Green 2016). Indeed, such functions have been tied to superior, middle, and inferior frontal regions, the inferior parietal cortex, the precuneus, and the basal ganglia in adults and/or children (Seeley et al. 2007; Mohades et al. 2014; Shen et al. 2019). All of these structures were implicated in the present study, either directly (in cortical grey matter measures) or indirectly (in white matter measures of connecting fibers). Thus, the observed bilingual/monolingual differences in developmental trajectories may be at least partly explained by group differences in executive functions as well as dorsal stream function and procedural memory.

However, the above circuits and functions might not fully explain the bilingual/monolingual developmental changes observed here. In particular, although the precuneus was found to show different developmental trajectories between the groups in all three cortical measures (thickness, volume, surface area), it is not importantly implicated in two of the three circuits and associated functions above, and is not generally implicated with a primary role in executive functions. So what might (additionally) explain its involvement here? The precuneus has been implicated in various functions, perhaps most notably aspects of visuo-spatial processing and declarative memory (Cavanna and Trimble 2006). Intriguingly, some evidence suggests that the precuneous underlies not only visuo-spatial processing, but also the linguistic processing of spatial relations (Wallentin et al. 2008), though it remains unclear why this function should be more engaged in the bilingual than monolingual developing brain. In contrast, declarative memory is clearly involved in both first and second language (working closely together with procedural memory), in particular for lexical knowledge, but also for various other aspects of language, including grammar and speech-sound representations (Ullman 2020; Ullman et al. 2020). Thus, learning, representing, and processing two (or more) languages may engage declarative memory more than one language does. This view is also consistent with the finding here that the posterior parietal and inferior temporal cortex, both of which play important roles in declarative memory and associated functions (Ullman 2016; Tagarelli et al. 2019), show bilingual/monolingual differences in their developmental trajectories. Nevertheless, it remains unclear why other declarative memory structures, in particular the hippocampus and other medial temporal lobe structures, were not involved here—though interestingly, the hippocampus has been found to be enlarged in adult second language learners (Mårtensson et al. 2012), remains plastic in active adult bilinguals (DeLuca et al. 2019b) and its volume declines at a slower rate in older bilinguals as compared to older monolinguals (Li et al. 2017; Voits et al. 2020).

Overall, the pattern of structures showing different developmental trajectories between the two groups seems largely consistent with prior studies of the bilingual brain. In adults, bilingual/monolingual differences have been found mainly in frontal (and the ACC), temporal, and parietal regions, as well as the basal ganglia (mainly the striatum) and thalamus, and a number of (mainly cortico-cortical) white matter tracts (see Sect. "Bilingualism and brain structure in adults"). In children, the small number of studies (which, however, focused on specific structures) have mainly implicated frontal and nearby cortex (IFG pars opercularis, middle frontal gyrus, the ACC), temporal cortex (STG), parietal cortex (inferior parietal), the basal ganglia (striatum), and tracts related to frontal cortex. The present study, which examined a large sample size across multiple structures and a range of measures, extends and further specifies our understanding of bilingual/monolingual differences in development. In particular, while it confirms the involvement of IFG pars opercularis, middle frontal gyrus, inferior parietal cortex, and (at least connections with) the basal ganglia in the developing bilingual brain, it suggests that certain other structures may also play differential roles in bilingual and monolingual development, in particular, though not only, in frontal and parietal regions.

### Implications, limitations, and conclusion

The study has a number of implications and limitations. The findings suggest that bilinguals’ and monolingual’s brains differ even during development. This clearly extends bilingual/monolingual brain differences from adults to the developing brain. The results suggest that the apparent resilience of the bilingual brain to aging (see introduction) may begin already during development, reinforcing the view that any beneficial effects of bilingualism on brain structure may require long-term experience using the two languages (Perani and Abutalebi 2015).

The finding that these patterns overlap to a fair extent with previously observed bilingual/monolingual brain differences in adults both validates the current findings with respect to the adult literature and suggests that some adult brain differences already emerge during childhood and adolescence. This is not in fact surprising given that the vast majority of studies of the adult bilingual brain examined bilinguals who learned more than one language prior to adulthood (Luk and Pliatsikas 2016; García-Pentón et al. 2016; Hayakawa and Marian 2019; Pliatsikas 2019). Nevertheless, the findings also indicate that during development, bilingual/monolingual differences are not exactly the same as those observed in adulthood. In particular, the results suggest that some bilingual/monolingual brain differences found during development disappear by early adulthood, while others appear soon thereafter. Indeed, we saw here that certain bilingual/monolingual differences that emerged by late childhood to early puberty (in particular, greater cortical thickness or volume for bilinguals in a number of regions, likely due to less grey matter loss during development) stabilized, reduced, or even disappeared by early adulthood. Additionally, certain findings that appear to be consistent in the adult literature in more experienced bilinguals were not found or were sparsely observed in the present study, in particular greater bilingual than monolingual subcortical and cerebellar volumes (not observed here) and greater white matter integrity (found in one tract).

Overall, these patterns seem in line with our predictions, namely that developing bilinguals without extensive experience should show greater cortical thickness and volume as compared to monolinguals, though these differences should then gradually decrease or disappear around the same time as they begin to show increased volumes for subcortical structures and greater white matter integrity. Interestingly, the lack of any bilingual/monolingual differences in subcortical volumes and the cerebellum, and the involvement of only one white matter tract, seems to be consistent with the finding that most cortical regions did not show full reconvergence between the groups by the oldest age in the sample, namely 21. These findings thus appear to jibe both with the broad adult literature (regarding both which structures are implicated and their changes over the course of bilingualism) and with our predictions for bilingual/monolingual development differences based in part on this literature.

Nevertheless, a number of limitations suggest the need for further studies. First, we did not predict the finding that at very early ages the bilinguals appeared to show lower values than monolinguals, indeed for nearly all metrics for all structures. It is not clear what may account for this pattern, though the relatively small sample size of bilinguals at these ages suggests treating this finding with caution. Second, the PING database did not contain detailed information regarding the ages of acquisition of bilinguals’ languages, nor the amount or type of language experience they had. The very early bilingual/monolingual effects suggest that the children at this age were exposed to two (or more) languages from an early point, indicating that at least a portion of the sample experienced early ages of acquisition. This also suggests the possibility—though this is speculative—that the rest of the sample might also have experienced quite early exposures to their languages, given that the older children and adolescents may have been drawn from similar subject pools at the same testing sites. This possibility is supported by the observed trajectories of different bilingual/monolingual patterns at different ages, which (as we have argued above) appear to be consistent with experience-based changes over time with similar ages of acquisition across the participants. Nevertheless, the age of exposure of the older children in this sample was not known, and thus future research should examine whether the findings here replicate with populations with clearly documented early ages of exposure, as well as investigating developmental trajectories with later exposures. Third, it may be argued that further measures against type I errors could have been taken. Indeed, we did not correct for multiple comparisons. This decision was taken due to the fact that our approach attempted to balance the likelihood of type I and type II errors, and was already conservative in that it was designed to reduce type I errors by virtue of an analytical procedure akin to a vibration of effects approach, with only those effects with significant results across all alternative analyses being reported. Additionally, the high degree of consistency in the observed patterns in the relative trajectories of bilingual and monolingual brain measures suggests that the reported findings are not spurious. Together, this suggests that type I errors are not prevalent in the findings reported here. Finally, we emphasize that the present study (like the vast majority of developmental studies across large age ranges) is cross-sectional, so caution is warranted in extrapolating to actual developmental patterns within subjects. Nevertheless, we suggest that our findings can provide a solid foundation for well-designed longitudinal developmental studies.

In sum, the present study suggests that bilinguals and monolinguals differ in quite consistent ways regarding the developmental trajectories of brain structures from early childhood to early adulthood. Thus, the study clearly extends previous observations of bilingual/monolingual brain differences in adults to development. The evidence presented here suggests that, as compared to monolinguals, bilinguals show more grey matter (less developmental loss) starting around late childhood and adolescence, mainly in frontal and parietal regions, as well as increased white matter integrity (greater developmental increase) starting in mid-late adolescence, specifically in fibers connecting the striatum and inferior frontal cortex. The findings not only suggest that there may be a developmental basis for some of the structural brain differences found between bilingual and monolingual adults, but also indicate that some bilingual/monolingual differences may occur in the developing but not the adult brain. Overall, the data indicate that the bilingual brain does indeed differ from the monolingual brain, and that this difference begins to be apparent even during development.

## Electronic supplementary material

Below is the link to the electronic supplementary material.

Supplementary file1 (DOCX 178 kb)

## Data Availability

Data obtained from a freely available dataset
