# Supplementary material for: The effect of bilingualism on brain development from early childhood to young adulthood
Source: Brain Struct Funct. 2020 Jul 20;225(7):2131–52. doi: 10.1007/s00429-020-02115-5 (PMC7473972; doi:10.1007/s00429-020-02115-5)
Supplement: Supplementary file 1 — Supplementary file1 (DOCX 178 kb) [file 429_2020_2115_MOESM1_ESM.docx]

**Supplementary Material**

**Contents:**

1. Longitudinal diffusivity results
2. Code for data analysis
3. **Longitudinal diffusivity results**

For details on the analytic approach, see Data analysis section in the paper.

No reliable effects involving bilingualism were obtained for transversal diffusivity for either first-level or second-level analyses for any white matter tract.

No reliable three-way interactions (Age x Bilingualism x Hemisphere) in first-level analyses were found for longitudinal diffusivity for any white matter tract. In second-level analyses (analysing both hemispheres together) four tracts yielded Age x Bilingualism interactions. See Table S1 and Figure S1.

Table S1: Two-way interactions and their follow-up analyses for longitudinal diffusivity

|  | | 2^nd^-level analysis | | | 3^rd^-level analyses | | | |  |
| --- | --- | --- | --- | --- | --- | --- | --- | --- | --- |
|  | |  | |  | Bilinguals | | Monolinguals | |  |
|  | | Bilingualism | | Age x Bilingualism | Age | | Age | |  |
| Inferior fronto-occipital fasciculus | ns | | ** | | | *** | | ns | |
| Uncinate fasciculus | ns | | * | | | ns | | *** | |
| Anterior thalamic radiation | ns | | * | | | ns | | *** | |
| Striatal—Inferior frontal cortex | ns | | * | | | * | | *** | |

Note. All edfs (estimated degrees of freedom) > 1. All Fs > 2.6. Only key effects are shown (Age was a reliable predictor for the inferior fronto-occipital fasciculus and the striatal—inferior frontal cortex tract). ns: not significant. Significance level: *** *p*s (in both alternate models for second level analyses) < 0.001; ** *p*s < 0.01; * *p*s < 0.05.


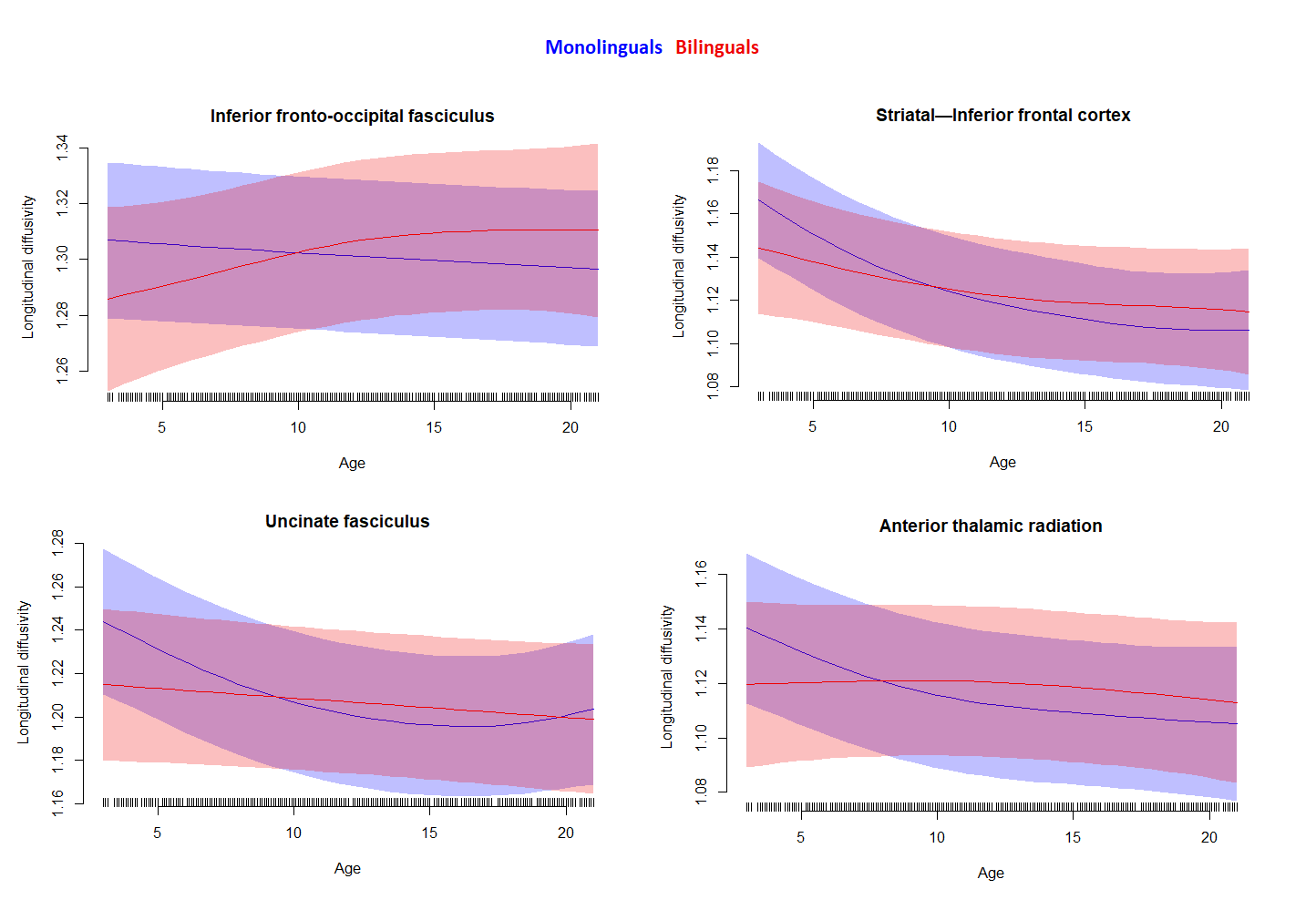


Figure S1. White matter tracts that yielded reliable age by bilingualism interactions for longitudinal diffusivity. That is, these tracts show significantly different developmental trajectories for longitudinal diffusivity between age 3 and 21 for bilinguals versus monolinguals. The developmental trajectories of bilinguals are shown in red, while the trajectories of monolinguals are shown in blue.

1. **Code for data analysis**

Data analysis with generalized additive models (GAMs)

Analysis of cortical thickness of the caudal anterior cingulate cortex is provided as an example. All structures and measures were analysed in this way. The only exceptions were the corpus callosum, forceps major, and forceps minor, which were not separated by hemisphere in the original data, and thus Model 1 was not run on these tracts.

1. Model 1, fitting smooths for Age, the Age x Bilingualism interaction, the Age x Hemisphere interaction, and the three-way Age x Bilingualism x Hemisphere interaction

library(mgcv)

library(itsadug)

dat.gam<-subset(dat.ct,MRILabel=="caudalanteriorcingulate")

# SubjID

dat.gam$SubjID <- as.factor(dat.gam$SubjID)

# Income and Education

dat.gam$Household_Income <- as.numeric(dat.gam$Household_Income)

dat.gam$Highest_Education <- as.numeric(dat.gam$Highest_Education)

## Create numerical predictors for Bilingualism and Hemisphere

## Note: NoBilingualism is Yes=0 and No=1; so Yes is reference level; etc.

dat.gam$YesBilingualism <- ifelse(dat.gam$Bilingualism=="Yes", 1, 0)

dat.gam$NoBilingualism <- ifelse(dat.gam$Bilingualism=="No", 1, 0)

## Note: RightHemisphere is lh=0 and rh=1; so lh is reference level; etc.

dat.gam$RightHemisphere <- ifelse(dat.gam$Hemisphere=="rh", 1, 0)

dat.gam$LeftHemisphere <- ifelse(dat.gam$Hemisphere=="lh", 1, 0)

## Create numerical predictors for interaction

dat.gam$NBxRH <- dat.gam$NoBilingualism*dat.gam$RightHemisphere

dat.gam$NBxLH <- dat.gam$NoBilingualism*dat.gam$LeftHemisphere

dat.gam$YBxRH <- dat.gam$YesBilingualism*dat.gam$RightHemisphere

dat.gam$YBxLH <- dat.gam$YesBilingualism*dat.gam$LeftHemisphere

## NUMERICAL FACTOR MODELS: REFERENCE + DIFFERENCE SMOOTH + 3-WAY INTERACTION

# Models with Age, Bilingualism, Hemisphere, all interactions (incl. 3-way), covariates

## Note: for the model specification we do *not* include Bilingualism and Hemisphere separately as main effects

## MODEL 1: NoBilingualism, RightHemisphere, NBxRH

m1 <- bam(GM_value ~ s(SubjID, bs="re") + s(DeviceSerialNumber, bs="re") + Household_Income + Highest_Education + GAF_europe + GAF_africa + GAF_amerind + GAF_eastAsia + GAF_oceania + GAF_centralAsia + s(Age) + s(Age, by=NoBilingualism) + s(Age, by=RightHemisphere) + s(Age, by=NBxRH), data=dat.gam, method="REML")

## MODEL 2: NoBilingualism, LeftHemisphere, NBxLH

m2 <- bam(GM_value ~ s(SubjID, bs="re") + s(DeviceSerialNumber, bs="re") + Household_Income + Highest_Education + GAF_europe + GAF_africa + GAF_amerind + GAF_eastAsia + GAF_oceania + GAF_centralAsia + s(Age) + s(Age, by=NoBilingualism) + s(Age, by=LeftHemisphere) + s(Age, by=NBxLH), data=dat.gam, method="REML")

## MODEL 3: YesBilingualism, RightHemisphere, YBxRH

m3 <- bam(GM_value ~ s(SubjID, bs="re") + s(DeviceSerialNumber, bs="re") + Household_Income + Highest_Education + GAF_europe + GAF_africa + GAF_amerind + GAF_eastAsia + GAF_oceania + GAF_centralAsia + s(Age) + s(Age, by=YesBilingualism) + s(Age, by=RightHemisphere) + s(Age, by=YBxRH), data=dat.gam, method="REML")

## MODEL 4: YesBilingualism, LeftHemisphere, YBxLH

m4 <- bam(GM_value ~ s(SubjID, bs="re") + s(DeviceSerialNumber, bs="re") + Household_Income + Highest_Education + GAF_europe + GAF_africa + GAF_amerind + GAF_eastAsia + GAF_oceania + GAF_centralAsia + s(Age) + s(Age, by=YesBilingualism) + s(Age, by=LeftHemisphere) + s(Age, by=YBxLH), data=dat.gam, method="REML")

1. Model 2, accounting for Bilingualism as an ordered factor and also including a smooth term for Age and a smooth term for the Age x Bilingualism interaction

library(mgcv)

library(itsadug)

dat.gam<-subset(dat.ct,MRILabel=="caudalanteriorcingulate")

# SubjID

dat.gam$SubjID <- as.factor(dat.gam$SubjID)

# Income and Education

dat.gam$Household_Income <- as.numeric(dat.gam$Household_Income)

dat.gam$Highest_Education <- as.numeric(dat.gam$Highest_Education)

## ORDERED FACTOR MODEL: REFERENCE + DIFFERENCE SMOOTH

## Note: for the model specification, we include both main effects of s(Age) and Bilingualism, and interaction

# Ordered factor for Bilingualism, and set contrasts. Ref= YES

dat.gam$Bilingualism <- as.factor(dat.gam$Bilingualism)

dat.gam$Bilingualism <- relevel(dat.gam$Bilingualism, ref="Yes")

dat.gam$OFBilingualism <- as.ordered(as.factor(dat.gam$Bilingualism))

contrasts(dat.gam$OFBilingualism) <- 'contr.treatment'

contrasts(dat.gam$OFBilingualism)

m.yes <- bam(GM_value ~ s(SubjID, bs="re") + s(DeviceSerialNumber, bs="re") +Household_Income + Highest_Education + GAF_europe + GAF_africa + GAF_amerind + GAF_eastAsia + GAF_oceania + GAF_centralAsia + OFBilingualism +s(Age) + s(Age, by=OFBilingualism), data=dat.gam, method="REML")

####################################################################

# Ordered factor for Bilingualism, and set contrasts. Ref= NO

dat.gam$Bilingualism <- as.factor(dat.gam$Bilingualism)

dat.gam$Bilingualism <- relevel(dat.gam$Bilingualism, ref="No")

dat.gam$OFBilingualism <- as.ordered(as.factor(dat.gam$Bilingualism))

contrasts(dat.gam$OFBilingualism) <- 'contr.treatment'

contrasts(dat.gam$OFBilingualism)

m.no <- bam(GM_value ~ s(SubjID, bs="re") + s(DeviceSerialNumber, bs="re") + Household_Income + Highest_Education + GAF_europe + GAF_africa + GAF_amerind + GAF_eastAsia + GAF_oceania + GAF_centralAsia + OFBilingualism + s(Age) + s(Age, by=OFBilingualism), data=dat.gam, method="REML")

1. Model 3, which includes a main effect of Bilingualism and a smooth for Age by each level of Bilingualism separately

library(mgcv)

library(itsadug)

dat.gam<-subset(dat.ct,MRILabel=="caudalanteriorcingulate")

# SubjID

dat.gam$SubjID <- as.factor(dat.gam$SubjID)

# Income and Education

dat.gam$Household_Income <- as.numeric(dat.gam$Household_Income)

dat.gam$Highest_Education <- as.numeric(dat.gam$Highest_Education)

dat.gam$Bilingualism <- as.factor(dat.gam$Bilingualism)

dat.gam$Bilingualism <- relevel(dat.gam$Bilingualism, ref="Yes")

## FACTOR MODEL: SEPARATE SMOOTHS

## Note: for the model specification, we do *not* include s(Age) separately

m0 <- bam(GM_value ~ s(SubjID, bs="re") + s(DeviceSerialNumber, bs="re") + Household_Income + Highest_Education + GAF_europe + GAF_africa + GAF_amerind + GAF_eastAsia + Bilingualism + s(Age, by=Bilingualism), data=dat.gam, method="REML")

plot_smooth(m0, view="Age", plot_all="Bilingualism", ylab = "Cortical Thickness (mm)", hide.label = TRUE, main = "Caudal anterior cingulate cortex", col = c("blue", "red2"))
